# *KCNQ1* Haplotypes Associate with Type 2 Diabetes in Malaysian Chinese Subjects

**DOI:** 10.3390/ijms12095705

**Published:** 2011-09-05

**Authors:** Riyadh Saif-Ali, Ikram S. Ismail, Zaid Al-Hamodi, Hesham M. Al-Mekhlafi, Lee C. Siang, Aied M. Alabsi, Sekaran Muniandy

**Affiliations:** 1 Department of Molecular Medicine, Faculty of Medicine, University of Malaya, Kuala Lumpur 50603, Malaysia; E-Mails: zalhamodi@yahoo.com (Z.A.-H.); bc_cslee@yahoo.com (L.C.S.); 2 Faculty of Medicine, Sana’a University, Sana’a, P.O. Box 13078, Yemen; E-Mail: halmekhlafi@yahoo.com; 3 Department of Medicine, Faculty of Medicine, University of Malaya Medical Center, University of Malaya, Kuala Lumpur 50603, Malaysia; E-Mail: ikram@um.edu.my; 4 Department of Parasitology, Faculty of Medicine, University of Malaya, Kuala Lumpur 50603, Malaysia; 5 Faculty of Agriculture and Biotechnology, Universiti Sultan Zainal Abidin, Kuala Terengganu 21030, Malaysia; E-Mail: aied@unisza.edu.my

**Keywords:** *KCNQ1*, SNPs, haplotype, diplotyps, type 2 diabetes

## Abstract

The aim of this study was to investigate the association of single nucleotide polymorphisms (SNPs) and haplotypes of potassium voltage-gated channel, KQT-like subfamily, member 1 (*KCNQ1*) with type 2 diabetes (T2D) in Malaysian Chinese subjects. The *KCNQ1* SNPs rs2237892, rs2283228 and rs2237895 were genotyped in 300 T2D patients and 230 control subjects without diabetes and metabolic syndrome. Two logistic regression models of analysis were applied, the first adjusted for age and gender while the second adjusted for age, gender and body mass index. The additive genetic analysis showed that adjusting for body mass index (BMI) even strengthened association of rs2237892, rs2283228 and rs2237895 with T2D (OR = 2.0, *P* = 5.1 × 10^−5^; OR = 1.9, *P* = 5.2 × 10^−5^; OR = 1.9, *P* = 7.8 × 10^−5^, respectively). The haplotype TCA containing the allele of rs2237892 (T), rs2283228 (C) and rs2237895 (A) was highly protective against T2D (Second model; OR = 0.17, *P* = 3.7 × 10^−11^). The *KCNQ1* rs2237892 (TT), and the protective haplotype (TCA) were associated with higher beta-cell function (HOMA-B) in normal subjects (*P* = 0.0002; 0.014, respectively). This study found that *KCNQ1* SNPs was associated with T2D susceptibility in Malaysian Chinese subjects. In addition, certain *KCNQ1* haplotypes were strongly associated with T2D.

## 1. Introduction

The *KCNQ1* gene has a total of 17 exons, spans 404 kb of chromosome sequence and is located on chromosome 11p15.5 [1]. *KCNQ1* codes for the pore-forming alpha subunit of the voltage-gated K+ channel (KvLQT1) that is highly expressed in the heart. This channel plays an important role in controlling repolarization of the ventricles [2]. *KCNQ1* is ubiquitously expressed in epithelial cells, including the endocrine and exocrine pancreatic cells [3]. *KCNQ1* was reported to be expressed in insulin-secreting cells, and inhibition of this potassium channel has been shown to significantly increase insulin secretion [4].

Genome wide association study (GWAS) has been applied to complex diseases, including T2D and has resulted in the identification of a growing number of trait susceptibility loci for T2D [5]. Two independent GWAS have identified *KCNQ1* as a novel T2D susceptibility gene in East Asian subjects [6,7]. More recently, two GWAS on Chinese Han and European populations confirmed *KCNQ1* as T2D susceptibility gene [8,9]. The association of T2D with *KCNQ1* variants was replicated in studies among Chinese [10–12], Singaporean [13,14], Indians [15], Pakistani [16] and in some Euro-Caucasians [6,17,18]. However, there is little data about the association of haplotypes of *KCNQ1* with T2D. The focus of this study was on the association of common variants of *KCNQ1* single nucleotide polymorphisms (SNPs) (rs2237892, rs2283228 and rs2237895), haplotypes and diplotypes with T2D in Malaysian Chinese subjects.

## 2. Results

Three hundred and forty-eight T2D and 354 control subjects who gave informed consent forms were recruited for this study. An application of the new metabolic syndrome criteria [19] on the control group resulted in 123 subjects with metabolic syndrome; therefore, they were excluded from the study. As a result of calculating % beta-cell insulin secretion using HOMA calculator, 3 diabetic and 1 normal subjects were excluded due to fasting insulin <20 pmol/L while 45 diabetic subjects were excluded due to fasting insulin >300 pmol/L. Consequently, 300 diabetic and 230 normal subjects without diabetes and metabolic syndrome were included in this study. The demography and biochemical parameters of the subjects are shown in Table 1.

### 2.1. Association of KCNQ1 SNPs with T2D

The SNPs included in this study did not deviate from the Hardy-Weinberg Equilibrium in the control group. The risk allele frequencies of rs2237892 (C), rs2283228 (A) and rs2237895 (C) in normal subjects were 0.69, 0.64 and 0.27 *versus* 0.78, 0.73 and 0.34 in diabetic patients, respectively. The first logistic regression model (adjusted for age and gender) showed that rs2237892, rs2283228, rs2237895 were associated with T2D (additive, OR = 1.6; 1.5; 1.5, *P* = 0.0005; 0.002; 0.004, respectively) (Table 2). Adjusting for body mass index (BMI) even strengthened the association of rs2237892, rs2283228, rs2237895 with T2D (additive, OR = 2.1; 1.9; 1.9, *P* = 5.1 × 10^−5^, 5.2 × 10^−5^, 7.8 × 10^−5^, respectively).

### 2.2. Association of KCNQ1 Haplotypes and Diplotypes with T2D

**Figure 1:**
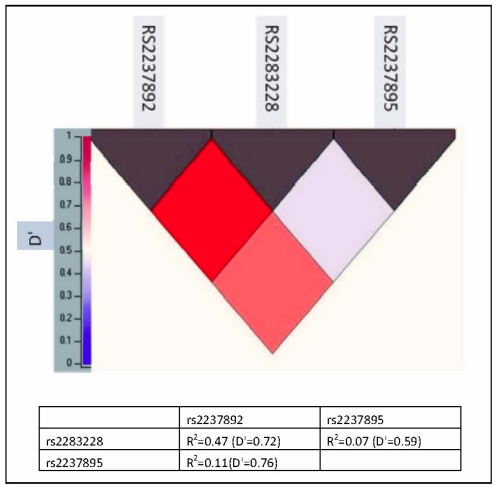
Pairwise linkage disequilibrium among *KCNQ1* single nucleotide polymorphisms (SNPs) in Malaysian Chinese. Values in the upper represent *KCNQ1* SNPs while values in the left represent D′ value.

Three-SNP haplotypes and diplotypes block were identified with significant linkage disequilibrium (LD). This block was constructed from rs2237892, rs2283228 and rs2237895 (Figure 1). The possible haplotype for each individual was adjusted to more than 0.5 resulting in 8 haplotypes and 23 diplotypes. The rare haplotypes (those below 2% frequency in cases or controls) were excluded from the analysis. Thus, 6 haplotypes and 8 diplotypes were further analyzed for their association with T2D.

The overall association of haplotypes with T2D was significant (*P* = 7.49 × 10^−6^). The haplotype TCA containing the protective alleles of rs2237892, rs2283228 and rs2237895 is more frequent in the normal (0.33) compared to diabetic subjects (0.15). Both logistic regression models showed that this haplotype was strongly protective against T2D (first model, OR = 0.33, *P* = 8.4 × 10^−7^; second model, OR = 0.17, *P* = 3.7 × 10^−11^) (Table 3). The haplotype CAC containing the risk allele of the SNPs included in this study was the most frequent haplotype (0.44 in normal subjects *vs.* 0.51 in diabetic subjects). Second logistic regression models showed that this haplotype was a risk for T2D (OR = 1.7, *P* = 0.008) whereas the first model showed this haplotype as borderline risk for T2D (OR = 1.4, *P* = 0.057).

The less frequent haplotype TAA (0.01 in normal *vs.* 0.06 in diabetic subjects was strongly associated with T2D (first model, OR = 4.6, *P* = 0.017; second model, OR = 6.0, *P* = 0.007). The results showed that haplotype CCC was significantly associated with T2D in the first logistic regression model (OR = 3.2, *P* = 0.021) whereas this significance was less evident in the second model (OR = 2.6, *P* = 0.083). Both logistic regression models showed that the haplotype CAA and CCA were no association with T2D.

The overall association of diplotypes with T2D was significant (*P* = 8.1 × 10^−7^). The diplotypes TCA-TCA and CAA-TCA containing the protective haplotype (TCA) showed a protection against T2D (first model, OR = 0.16, *P* = 8 × 10^−5^; OR = 0.4, *P* = 0.0003, respectively) (second model, OR = 0.13, *P* = 4.4 × 10^−5^; OR = 0.09, *P* = 2.9 × 10^−10^, respectively) (Table 4). In addition, the second logistic regression model showed that the diplotype CAC-CAC containing the risk haplotype (CAC) was strongly a risk for T2D (OR = 3.9, *P* = 0.008) and diplotype CCA-CAC was a borderline risk for T2D (OR = 2.6, *P* = 0.07) whereas the first model did not show such effect. Both logistic regression models showed that the other diplotypes (CAA-CAC, CAA-CAA, TCA-CAC and CAA-CCA) were not significantly associated with T2D.

### 2.3. Impact of KCNQ1 SNPs, Haplotypes and Diplotypes on Beta-Cell Function in Normal Subjects

Two general linear models (GLM), the first adjusted for age and gender and the second adjusted for age, gender and BMI were approached to identify potential mediators who link the KCN Q1 variants, with T2D. The three SNPs were tested for their associations with diabetes-related quantitative traits, beta cell function (HOMA-B). Both GLM models showed that HOMA-B in normal subjects those had the variant of KCNQ1 rs2237892 (TT) was higher than CC and CT genotype (*P* = 0.002, 0.0002, respectively) (Table 5). The second GLM showed little effect of rs2283228 CC genotype on HOMA-B (*P* = 0.034) compared to other genotypes of this SNP. The results showed that rs2237895 had no effect on beta-cell function.

Three haplotypes (CAA, CAC and TCA) and 5 diplotypes (CAA-CAA, CAA-CAC, CAA-TCA, TCA-CAC and TCA-TCA) fitted the criteria of parametric analysis (count ≥ 25) to evaluate their impact on the beta-cell function. Both GLM showed that HOMA-B was higher in normal subjects, which had the protective haplotype TCA than those normal subjects carrying the risk haplotyype CAC or CAA (*P* = 0.003; 0.014, respectively). Furthermore, the two protective diplotype (TCA-TCA, and CAA-TCA) showed a higher HOMA-B than the other diplotypes (first GLM, *P* = 1.5 × 10^−6^; second GLM, *P* = 5.7 × 10^−6^).

## 3. Discussion

The association of *KCNQ1* SNPs, haplotypes and diplotypes with T2D among Malaysian Chinese was studied. Common variants of *KCNQ1* SNPs rs2237892, rs2283228 and rs2237895 were selected for this study based on the Unoki and Yasuda findings [6,7] that these SNPs showed an association with T2D in Asian populations. The present study found that the common *KCNQ1* SNPs rs2237892, rs2237895 and rs2283228 were strongly associated with T2D, which is in agreement with previous reports [6–15,18,20,21]. Adjusting for BMI even strengthened the association of *KCNQ1* variants with T2D. The odds ratios of the second logistic regression model were 1.9–3.7 (additive genetic analysis) which are higher than the previous reported odds ratios (1.2–1.6) [6–15,18,20,21].

**Figure 2:**
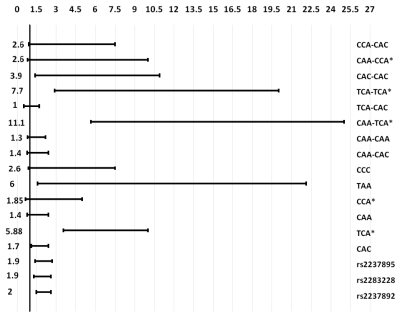
Odds ratios and 95% confidence intervals of the association of individual SNPs (additive genetic models), haplotypes and diplotypes with T2D. Left number, odds ratio; bar, 95% confidence interval; * reciprocal odds ratio and 95% confidence interval were represented.

The haplotypes and diplotypes showed a higher association with T2D than single individual SNPs did. Plotting the second logistic regression odds ratios and 95% CI of the association of SNPs (additive genetic models), haplotypes and diplotypes with T2D resulted in, the reciprocal 95% CI of the more associated haplotype (TAA) and diplotypes (CAA-TCA and TCA-TCA) were higher than 95% CI of individual SNPs, and were not overlapping each other (Figure 2). Other haplotype blocks have been reported to be associated with T2D [11,12,15].

The current study confirmed the association of the *KCNQ1* variants with impaired b-cell function estimated by HOMA-B, and the risk alleles of rs2237892 and rs2283228 were significantly associated with lower HOMA-B values. This finding is in agreement with a previous report [12]. The increased risk for T2D linked to *KCNQ1* gene is likely to be caused by a reduction in insulin secretion [10–13]. The pore-forming alpha subunit of the voltage-gated K+ channel (KvLQT1) (encoded by *KCNQ1*) and the regulatory beta subunit ISK (encoded by potassium channel, voltage-gated, ISK-related subfamily, member 1; *KCNE1* gene) co-assemble to form the I_(KS)_ potassium channel in the pancreas [22]. Intrinsically, there is a possibility that *KCNQ1* polymorphisms alter the role of the I_(KS)_ potassium channel, causing decreased insulin secretion, leading in time to T2D [13]. However, homozygous Kcnq1^−/−^ mice have been reported not to show hyperglycaemia or glucose intolerance, and the contribution of the kcnq1-encoded protein to the molecular pathogenesis of T2D remains unclear [6]. A recent study, found that both blood glucose and insulin levels were lower in kcnq1^−/−^ than in kcnq1^+/+^mice and the uptake of glucose into skeletal muscle, liver, kidney and lung tissue was significantly higher in kcnq1^−/−^ than inkcnq1^+/+^mice [23] leading to a suggestion that kcnq1 is a novel molecule affecting insulin sensitivity.

## 4. Materials and Methods

### 4.1. Subjects and Data Collection

T2D Malaysian Chinese subjects aged between 30 and 70 years who attended the University Malaya Medical Centre (UMMC), Kuala Lumpur for treatment were randomly approached and asked to participate voluntarily in this study (target group). For the control group, the physically normal Malaysian Chinese subjects who attended the UMMC for routine medical check-ups were approached. The study was approved by the Medical Ethics Committee of the University of Malaya Medical Centre. Venous Blood (10 mL) was collected from each subject after obtaining written consent.

### 4.2. Biochemical Analyses

Glucose, triacylglycerol, total cholesterol and HDLc were measured by an automated analyzer (Dimension^®^ RxL Max^®^ Integrated Chemistry System), and insulin was measured by ADVIA Centaur assay XP Immunoassay System (Siemens Healthcare Diagnostics Inc. Deerfield, IL USA). % beta-cell insulin secretion (HOMA-B) was calculated using the Homeostasis Model Assessment (HOMA2) Calculator v2.2, which is available online from Oxford Center for Diabetes, Endocrinology and Metabolism.

### 4.3. Genetic Analyses

Single nucleotide polymorphisms of *KCNQ1*; rs2237892, rs2283228 and rs2237895 were selected for genotypic analysis in Malaysian T2D subjects based on the findings of Unoki and Yasuda [6,7] that these SNPs are associated with T2D in Asian populations. The SNPs sequences were obtained from the database of the US National Library of Medicine [24]. Specific primers were designed for each SNP by FastPCR program. DNA extraction was achieved through the salt precipitation method. All SNPs were amplified using a 96 microwell plate StepOnePlus thermocycler (Applied Biosystems Inc, Foster City, USA). The SNPs rs2237892, rs2283228 and rs2237895 were genotyped by restriction enzymes BsoBI, BstNI and SmaI, respectively. Polyacrylamide gel electrophoresis (7%) was used for detection the digested product of the PCR amplicons. The Polyacrylamide gel was stained by 0.1 μg/mL ethidium bromide for 5 minutes and then visualized by exposure to ultraviolet light in the gel imaging system (Infinity 3026, Vilber Lourmat, Marnela Valled, France). To confirm the restriction enzyme results, approximately 10% of each SNPs PCR amplicons (54 samples) was sequenced by automated DNA sequencer (3130xl Genetic Analyzer, Applied Biosystems, Foster City, CA, USA) using terminator cycle sequencing kit v3.1 (Applied Biosystems). The sequencing results were identical to the restriction enzymes’ results of rs2237892 and rs2237895 (kappa = 1) where the kappa was 0.932 for rs2283228.

### 4.4. Statistical Analysis

HelixTree 7.0 SNP and Variation Suite for Genetic Statistics (SVS) was used to study the linkage disequilibrium (LD) between SNP and construct haplotypes and diplotypes of related SNPs. The deviation from the Hardy-Weinberg Equilibrium was tested by De Finetti program [25]. The other statistical analyses were performed on SPSS version 11.5. Two logistic regression models were applied for the evaluation of associations of the *KCNQ1* SNPs, recessive, dominant and additive genetic analysis and the association of haplotypes and diplotypes with T2D. The first model was adjusted for age and gender while the second model, adjusted for age, gender and body mass index.

The overall association of haplotypes and diplotypes with T2D was evaluated by crosstabs (chi-square test). The impact of the *KCNQ1* SNPs variants, haplotypes and diplotypes on beta-cell insulin secretion (HOMA-B), was evaluated by two general linear models (GLM). The first adjusted for age and gender while the second adjusted for age, gender and BMI. HOMA-B values were skewed and, therefore, normalized by logarithmic transformation. Means were subsequently back transformed for presentation as geometric means.

## 5. Conclusions

This study showed that *KCNQ1* common variants were associated with T2D in Malaysian Chinese subjects. In addition, analysis of *KCNQ1* haplotypes and diplotypes supported the association of *KCNQ1* gene polymorphisms with T2D. Furthermore, *KCNQ1* SNPs, haplotypes and diplotypes were associated with beta-cell function in normal subjects without diabetes and metabolic syndrome.

## Figures and Tables

**Table 1:** Demography and biochemical parameters.

Parameters		Normal *n* = 230	Type 2 diabetes *n* = 300	*P*-value
Gender	Male %	61.3	51	
	Female %	38.7	49	

Family history of diabetes %	Male %	32	76	
	Female %	34	63	

Age (years)		52.9 ± 9.15	49.8 ± 7.42	<0.001
Height (m)		1.62 ± 0.08	1.63 ± 0.09	0.18
Weight (kg)		63.1 ± 15.5	75.0 ± 15.2	<0.001
BMI(kg/m^2^)		24.1 ± 5.14	28.3 ± 5.15	<0.001
Waist (cm)		84.7 ± 13.1	95.7 ± 12.3	<0.001
Hip (cm)		99 ± 10.1	104 ± 10.1	0.001
Waist/Hip		0.85 ± 0.07	0.92 ± 0.07	<0.001
Systolic blood pressure		136 ± 18.5	136 ± 18.5	0.97
		81 ± 9.7	82 ± 10.5	0.20
Fasting insulin (pmol/L)		63.8 ± 44.6	103 ± 56.6	<0.001
Fasting glucose (mmol/L)		5.1 ± 0.49	8.3 ± 2.74	<0.001
Triacylglycerol (mmol/L)		1.1 ± 0.44	2.1 ± 1.20	<0.001
HDL cholesterol (mmol/L)		1.52 ± 0.32	1.21 ± 0.28	<0.001
Insulin resistance (IR)		1.4 ± 0.93	2.5 ± 1.44	<0.001

**Table 2:** Association of *KCNQ1* single nucleotide polymorphisms with type 2 diabetes evaluated by recessive, dominant and additive genetic models.

					Recessive model		Dominant model		Additive model	
			
NPs	Group	Genotype *n* (distribution %)			OR (95% CI)	*P*-value	OR (95% CI)	*P*-value	OR (95% CI)	*P*-value
First logistic regression model, analysis adjusted for age and gender										
rs2237892		TT	CT	CC	1.9 (1.29–2.66)	**0.001**	2.1 (1.09–3.94)	**0.026**	1.6 (1.25–2.18)	**0.0005**
	normal	27(11.7)	90(39.1)	113(49.1)						
	diabetic	18(6.0)	99(33.0)	183(61.0)						
rs2283228		CC	AC	AA	1.8 (1.24–2.52)	**0.002**	1.7 (0.97–2.93)	0.06	1.5 (1.17–1.97)	**0.002**
	normal	33(14.3)	98(42.6)	99(43.0)						
	diabetic	27(9.0)	105(35.0)	168(56.0)						
rs2237895		CC	AC	AA	1.6 (0.83–3.17)	0.16	1.7 (1.17–2.39)	**0.004**	1.5 (1.14–2.01)	**0.004**
	normal	14(6.1)	96(41.7)	120(52.2)						
	diabetic	30(10.0)	147(49.0)	123(41.0)						

Second logistic regression model, analysis adjusted for age, gender, and body mass index										
rs2237892 *		TT	CT	CC	2.2 (1.5–3.4)	**0.0002**	2.7 (1.3–5.5)	**0.007**	2.0 (1.4–2.7)	**5.1 × 10^−5^**
	normal	27(12.6)	81(37.7)	107(49.8)						
	diabetic	18(6.0)	99(33.0	183(61.0)						
rs2283228 *		CC	AC	AA	2.3 (1.5–3.5)	**7.3 × 10^−5^**	2.2 (1.2–4.1)	**0.011**	1.9 (1.4–2.5)	**5.2 × 10^−5^**
	normal	33(14.9)	89(40.3)	99(44.8)						
	diabetic	27(9.0)	105(35.0)	168(56.0)						
rs2237895 *		CC	AC	AA	3.7 (1.7–8.1)	**0.001**	2.0 (1.3–3.0)	**0.001**	1.9 (1.4–2.7)	**7.8 × 10^−5^**
	normal	11(5.0)	96(44.0)	111(50.9)						
	diabetic	30(10.0)	147(49.0)	123(41.0)						

In the additive model, genotype of homozygote for the non-risk allele (0/0), heterozygote (1/0) and homozygote for the risk allele (1/1 were coded as 0, 1 and 2 respectively). The recessive model was defined as 1/1 *vs.* 1/0 + 0/0 and dominant model as 1/1+1/0 vs 0/0;

* The outlier (studentized residual is greater than 2.0 or less than −2.0) were excluded.

**Table 3:** Association of common haplotypes with type 2 diabetes.

Haplotypes rs2237892, rs2283228, rs2237895	Frequency					
	Normal *n* (230)	Type 2 diabetes *n* (300)	Odds ratio	95% CI	*P*-Value	Overall *P*-value
First logistic regression model, analysis adjusted for age and gender						
CAC	0.44	0.51	1.4	0.99–2.01	0.057	7.49 × **10^−6^**^a^
TCA	0.33	0.15	0.33	0.21–0.51	**8.4 × 10^−7^**	
CAA	0.13	0.16	1.3	0.86–2.21	0.263	
CCA	0.05	0.04	0.7	0.30–1.63	0.41	
TAA	0.01	0.06	4.6	1.31–16.19	**0.017**	
CCC	0.02	0.07	3.2	1.20–8.81	**0.021**	

Second logistic regression model, analysis adjusted for age, gender and body mass index						
CAC *	0.44	0.51	1.7	1.1–2.4	**0.008**	
TCA *	0.34	0.15	0.17	0.1–0.28	**3.7 × 10^−11^**	
CAA *	0.14	0.16	1.4	0.79–2.4	0.26	
CCA *	0.05	0.04	0.54	0.2–1.5	0.23	
TAA *	0.01	0.06	6.0	1.6–22.1	**0.007**	
CCC *	0.02	0.07	2.6	0.88–7.5	0.083	

* The outlier (studentized residual is greater than 2.0 or less than −2.0) were excluded;

a non-adjusted overall *P*-value.

**Table 4:** Association of common diplotypes with type 2 diabetes.

Diplotypes rs2237892, rs2283228, rs2237895	(Frequency)					
	Normal *n* (230)	Type 2 diabetes *n* (300)	Odds ratio	95% CI	*P*-Value	Overall *P*-value
First logistic regression model, analysis adjusted for age and gender						
CAA-CAC	0.22	0.25	1.3	0.86–1.99	0.21	8.1 × **10^−7^**^a^
CAA-CAA	0.13	0.16	1.3	0.81–2.21	0.26	
CAA-TCA	0.18	0.09	0.4	0.21–0.63	**0.0003**	
TCA-CAC	0.13	0.12	0.92	0.54–1.57	0.77	
TCA-TCA	0.12	0.02	0.16	0.06–0.40	**8 × 10^−5^**	
CAC-CAC	0.03	0.06	2.2	0.85–5.74	0.104	
CAA-CCA	0.05	0.03	0.48	0.19–1.19	0.11	
CCA-CAC	0.04	0.06	1.8	0.78–4.10	0.17	

Second logistic regression model, analysis adjusted for age, gender, and body mass index						
CAA-CAC *	0.23	0.25	1.4	0.79–2.4	0.26	
CAA-CAA *	0.14	0.16	1.3	0.82–1.3	0.26	
CAA-TCA *	0.19	0.08	0.09	0.04–0.18	**2.9 × 10^−10^**	
TCA-CAC *	0.14	0.12	1.0	0.54–1.7	0.88	
TCA-TCA *	0.12	0.02	0.13	0.05–0.34	**4.4 × 10^−5^**	
CAC-CAC *	0.01	0.06	3.9	1.4–10.9	**0.008**	
CAA-CCA *	0.05	0.03	0.38	0.12–1.2	0.09	
CCA-CAC *	0.03	0.06	2.6	0.92–7.5	0.07	

* The outlier (studentized residual is greater than 2.0 or less than −2.0) were excluded;

a , non-adjusted overall *P*-value.

**Table 5:** 3 Impact of *KCNQ1*, haplotypes and diplotypes on beta-cell function (HOMA-B) in normal subjects.

	Non adjusted HOMA-B means(CI)	Adjusted for age, gender			Adjusted for age, gender, BMI		

		HOMA-B mean(CI)	Parameter estimate *P*-value	Univariate *P*-value	HOMA-B Mean(CI)	Parameter estimate *P*-value	Univariate *P*-value
rs2237892							

CC(n = 113)	96(90–103)	98(92–105)	0.006	0.002	100(95–106)	0.01	0.0002
CT(n = 90)	94(87–101)	92(85–98)	0.0004		90(85–96)	0.0003	
TT(n = 27) Ref	120(104–137)	121(106–138)			118(105–132)		

rs2283228							

AA(99)	97(90–104)	97(90–104)	0.33	0.584	102(96–109)	0.80	0.034
AC(98)	97(87–101)	97(90–103)	0.30		92(86–97)	0.039	
CC(33) Ref	104(92–118)	104(92–118)			104(94–115)		

rs2237895							

CC(14)	100(83–122)	98(81–119)	0.91	0.74	106(90–125)	0.36	0.554
AC(96)	95(88–102)	96(89–103)	0.44		96(91–103)	0.70	
AA(120) Ref	100(94–107)	99(93–106)			98(93–104)		

haplotypes							

CAA(n = 30)	84(74–95)	85(75–97)	0.001	0.003	90(81–100)	0.007	0.014
CAC(n = 102)	97(91–104)	97(91–104)	0.026		97(92–103)	0.026	
TCA(n = 75) Ref	111(103–120)	110(102–119)			107(101–115)		

Diplotype							

CAA-CAA(n = 30)	84(75–94)	85(76–95)	4.0 × 10^−5^	1.5 × 10^−6^	90(81–99)	0.0001	5.7 × 10^−6^
CAA-CAC(n = 51)	106(97–115)	107(98–116)	0.12		110(102–119)	0.20	
CAA-TCA(n = 42)	117(107–129)	114(104–126)	0.55		105(96–115)	0.062	
TCA-CAC(n = 30)	83(75–93)	84(75–94)	1.9 × 10^−5^		85(77–94)	5.8 × 10^−6^	
TCA-TCA(n = 27) Ref	120(106–134)	120(107–135)			120(108–133)		

% beta-cell insulin secretion was log-transformed before analyses, and the data were presented as geometric means; Ref, reference, the protective genotype, haplotypes and diplotypes were selected to be a reference for the comparison; CI, confidence interval.

## References

[1] Neyroud N, Richard P, Vignier N, Donger C, Denjoy I, Demay L, Shkolnikova M, Pesce R, Chevalier P, Hainque B (1999). Genomic organization of the KCNQ1 K^+^ channel gene and identification of C-terminal mutations in the long-QT syndrome. Circ Res.

[2] Barhanin J, Lesage F, Guillemare E, Fink M, Lazdunski M, Romey G (1996). K_V_LQT1 and lsK (minK) proteins associate to form the *I*_KS_cardiac potassium current. Nature.

[3] Thevenod F (2002). Ion channels in secretory granules of the pancreas and their role in exocytosis and release of secretory proteins. Am J Physiol Cell Physiol.

[4] Ullrich S, Su J, Ranta F, Wittekindt OH, Ris F, Rosler M, Gerlach U, Heitzmann D, Warth R, Lang F (2005). Effects of IKs channel inhibitors in insulin-secreting INS-1 cells. Pflüg Arch Eur J Physiol.

[5] Prokopenko I, McCarthy MI, Lindgren CM (2008). Type 2 diabetes: New genes, new understanding. Trends Genet.

[6] Unoki H, Takahashi A, Kawaguchi T, Hara K, Horikoshi M, Andersen G, Ng DP, Holmkvist J, Borch-Johnsen K, Jorgensen T (2008). SNPs in KCNQ1 are associated with susceptibility to type 2 diabetes in East Asian and European populations. Nat Genet.

[7] Yasuda K, Miyake K, Horikawa Y, Hara K, Osawa H, Furuta H, Hirota Y, Mori H, Jonsson A, Sato Y (2008). Variants in KCNQ1 are associated with susceptibility to type 2 diabetes mellitus. Nat Genet.

[8] Tsai FJ, Yang CF, Chen CC, Chuang LM, Lu CH, Chang CT, Wang TY, Chen RH, Shiu CF, Liu YM (2010). A genome-wide association study identifies susceptibility variants for type 2 diabetes in Han Chinese. PLoS Genet.

[9] Voight BF, Scott LJ, Steinthorsdottir V, Morris AP, Dina C, Welch RP, Zeggini E, Huth C, Aulchenko YS, Thorleifsson G (2010). Twelve type 2 diabetes susceptibility loci identified through large-scale association analysis. Nat Genet.

[10] Hu C, Wang C, Zhang R, Ma X, Wang J, Lu J, Qin W, Bao Y, Xiang K, Jia W (2009). Variations in KCNQ1 are associated with type 2 diabetes and beta cell function in a Chinese population. Diabetologia.

[11] Liu Y, Zhou DZ, Zhang D, Chen Z, Zhao T, Zhang Z, Ning M, Hu X, Yang YF, Zhang ZF (2009). Variants in KCNQ1 are associated with susceptibility to type 2 diabetes in the population of mainland China. Diabetologia.

[12] Qi Q, Li H, Loos RJ, Liu C, Wu Y, Hu FB, Wu H, Lu L, Yu Z, Lin X (2009). Common variants in KCNQ1 are associated with type 2 diabetes and impaired fasting glucose in a Chinese Han population. Hum Mol Genet.

[13] Tan JT, Nurbaya S, Gardner D, Ye S, Tai ES, Ng DP (2009). Genetic variation in KCNQ1 associates with fasting glucose and beta-cell function: a study of 3,734 subjects comprising three ethnicities living in Singapore. Diabetes.

[14] Tan JT, Ng DP, Nurbaya S, Ye S, Lim XL, Leong H, Seet LT, Siew WF, Kon W, Wong TY (2010). Polymorphisms identified through genome-wide association studies and their associations with type 2 diabetes in Chinese, Malays, and Asian-Indians in Singapore. J Clin Endocrinol Metab.

[15] Been LF, Ralhan S, Wander GS, Mehra NK, Singh JR, Mulvihill JJ, Aston CE, Sanghera DK (2011). Variants in KCNQ1 increase type II diabetes susceptibility in South Asians: A study of 3,310 subjects from India and the US. BMC Med Genet.

[16] Rees SD, Hydrie MZ, Shera AS, Kumar S, O’Hare JP, Barnett AH, Basit A, Kelly MA (2011). Replication of 13 genome-wide association (GWA)-validated risk variants for type 2 diabetes in Pakistani populations. Diabetologia.

[17] Holmkvist J, Banasik K, Andersen G, Unoki H, Jensen TS, Pisinger C, Borch-Johnsen K, Sandbaek A, Lauritzen T, Brunak S (2009). The type 2 diabetes associated minor allele of rs2237895 KCNQ1 associates with reduced insulin release following an oral glucose load. PLoS One.

[18] Jonsson A, Isomaa B, Tuomi T, Taneera J, Salehi A, Nilsson P, Groop L, Lyssenko V (2009). A variant in the KCNQ1 gene predicts future type 2 diabetes and mediates impaired insulin secretion. Diabetes.

[19] Alberti KG, Eckel RH, Grundy SM, Zimmet PZ, Cleeman JI, Donato KA, Fruchart JC, James WP, Loria CM, Smith SC (2009). Harmonizing the metabolic syndrome: A joint interim statement of the International Diabetes Federation Task Force on Epidemiology and Prevention; National Heart, Lung, and Blood Institute; American Heart Association; World Heart Federation; International Atherosclerosis Society; and International Association for the Study of Obesity. Circulation.

[20] Xu M, Bi Y, Xu Y, Yu B, Huang Y, Gu L, Wu Y, Zhu X, Li M, Wang T (2010). Combined effects of 19 common variations on type 2 diabetes in Chinese: results from two community-based studies. PLoS One.

[21] Lee YH, Kang ES, Kim SH, Han SJ, Kim CH, Kim HJ, Ahn CW, Cha BS, Nam M, Nam CM (2008). Association between polymorphisms in SLC30A8, HHEX, CDKN2A/B, IGF2BP2, FTO, WFS1, CDKAL1, KCNQ1 and type 2 diabetes in the Korean population. J Hum Genet.

[22] Warth R, Garcia Alzamora M, Kim JK, Zdebik A, Nitschke R, Bleich M, Gerlach U, Barhanin J, Kim SJ (2002). The role of KCNQ1/KCNE1 K^+^ channels in intestine and pancreas: lessons from the KCNE1 knockout mouse. Pflüg Arch Eur J Physiol.

[23] Boini KM, Graf D, Hennige AM, Koka S, Kempe DS, Wang K, Ackermann TF, Foller M, Vallon V, Pfeifer K (2009). Enhanced insulin sensitivity of gene-targeted mice lacking functional KCNQ1. Am J Physiol Regul Integr Comp Physiol.

[24] The United States National Library of Medicine http://www.ncbi.nlm.nih.gov/snp.

[25] Institute of Human Genetics, Technical University Munich.

